# X-Linked Duchenne-Type Muscular Dystrophy in Jack Russell Terrier Associated with a Partial Deletion of the Canine *DMD* Gene

**DOI:** 10.3390/genes11101175

**Published:** 2020-10-08

**Authors:** Barbara Brunetti, Luisa V. Muscatello, Anna Letko, Valentina Papa, Giovanna Cenacchi, Marco Grillini, Leonardo Murgiano, Vidhya Jagannathan, Cord Drögemüller

**Affiliations:** 1 Department of Veterinary Medical Sciences, University of Bologna, 40064 Bologna, Italy; luisaver.muscatello2@unibo.it; 2 Institute of Genetics, Vetsuisse Faculty, University of Bern, 3001 Bern, Switzerland; anna.letko@vetsuisse.unibe.ch (A.L.); leomur@vet.upenn.edu (L.M.); vidhya.jagannathan@vetsuisse.unibe.ch (V.J.); cord.droegemueller@vetsuisse.unibe.ch (C.D.); 3 Department of Biomedical and Neuromotor Sciences, University of Bologna, 40138 Bologna, Italy; valentina.papa2@unibo.it (V.P.); giovanna.cenacchi@unibo.it (G.C.); 4 Pathology Unit, S Orsola Malpighi Hospital, University of Bologna, 40138 Bologna, Italy; marco.grillini@aosp.bo.it; 5 Department of Clinical Sciences & Advanced Medicine, School of Veterinary Medicine, University of Pennsylvania, Philadelphia, PA 19104, USA

**Keywords:** canine, dystrophinopathy, Duchenne, immunohistochemistry, precision medicine

## Abstract

A 9-month old male Jack Russell Terrier started showing paraparesis of the hindlimbs after a walk. Hospitalized, the dog went into cardiac arrest, and later died. Necroscopic examination revealed a severe thickness of the diaphragm, esophagus, and base of the tongue, leading to the diagnosis of muscular dystrophy. The histology confirmed the marked size variation, regeneration, and fibrosis replacement of the skeletal muscle fibers. Immunohistochemistry demonstrated the absence of dystrophin confirming the diagnosis. Transmission electron microscopy showed disarrangement of skeletal muscle fibers. Finally, whole-genome sequencing identified a ~368kb deletion spanning 19 exons of the canine dystrophin (*DMD*) gene. This pathogenic loss-of-function variant most likely explains the observed disease phenotype. The X-chromosomal variant was absent in seven controls of the same breed. Most likely, this partial deletion of the *DMD* gene was either transmitted on the maternal path within the family of the affected dog or arose de novo. This study revealed a spontaneous partial deletion in *DMD* gene in a Jack Russell Terrier showing a Duchenne-type muscular dystrophy due to non-functional dystrophin.

## 1. Introduction

Duchenne and Becker muscular dystrophies are X-linked recessive disorders, therefore these forms occur predominantly in males. They are caused by genetic variants in the dystrophin gene (*DMD*), the largest gene in the human genome, and as such are called dystrophinopathies [1]. The *DMD* gene spanning 2.4 Mb on the X chromosome encodes 79 exons for a 427-kD protein, called dystrophin, a rod-shaped protein located on the inner face of the plasma membrane of all types of myofibers and anchors cytoskeletal F-actin to the extracellular matrix protein laminin [2,3]. Dystrophin protein has four main functional domains. The N-terminus is the cysteine-rich actin-binding domain, while the carboxy-terminal domain interacts with β-dystroglycan as well as with dystrobrevin and syntrophin. The central rod domain comprises the majority of the mass of the dystrophin molecule, forming a flexible, rod-shaped structure [4]. In humans, a causative genetic variant in *DMD* was found in 96% of Duchenne muscular dystrophy (DMD) cases and 82% of Becker muscular dystrophy (BMD) cases. Around one third of pathogenic variants in *DMD* are spontaneously occurring de novo mutations in the affected male patients [1]. The most common genetic variants within *DMD* are large deletions (approximately 70%) or duplications (10–14%) often encompassing numerous exons of the gene [1]. 

Mutations may occur throughout the 79 exons of the *DMD* gene but concentrate in major (exons 45–53) and minor (exons 2–20) hotspot areas. According to Leiden’s database, ~40% of *DMD* gene variants are deletions of a mean length of 6.5 exons, with exon 47 being most commonly affected [5]. These deletions tend to predominate in one of two hotspots, namely, the central rod domain around exons 44–53 (~80%) and, to a lesser extent (~20%), at the 5′-end of the gene [6,7]. Duplications occur most frequently in the region of exon 20 [5]. Dystrophin is important for the maintenance of the structural integrity of muscle fibers during contraction. Both Duchenne- and Becker-types of muscular dystrophies have similar signs and symptoms and are due to different mutations in the same gene. In human Duchenne muscular dystrophy, variants affecting the *DMD* gene result in a severely truncated, non-functional dystrophin, while in Becker muscular dystrophy, the mutations result in a truncated semi-functional dystrophin protein [8]. The two conditions differ in their severity, age of onset, and rate of progression. The signs of BMD are usually milder and more variable [9].

The diagnostic evaluation of muscular dystrophy often includes a muscle biopsy to demonstrate fibrosis, muscle fiber size variation, internalization of muscle nuclei, and more importantly, the abnormal expression of dystrophin by immunostaining or Western blot analysis. Fibro-fatty replacement, inflammation, and degenerative fibers are commonly described histological features [10]. Similar muscular dystrophies, homologous to human DMD and BMD, also occur in dogs (OMIA 001081-9615), cats (OMIA 001081-9685), pigs (OMIA 001081-9823), and mice [9]. Thus far, 12 different causative *DMD* variants for DMD have been described in several dog breeds (OMIA 001081-9615). For instance, the Golden Retriever muscular dystrophy model is widely used as a useful animal model, because these severely affected dogs show a phenotype strongly resembling human DMD on a clinical and histopathological level [10].

## 2. Materials and Methods

### 2.1. Ethics Statement

The dog in this study was privately owned, and samples were collected during the necropsy requested from the owner because the dog died unexpectedly.

### 2.2. Clinical History and Necropsy Request 

A 9-month old male Jack Russell Terrier started showing paraparesis of the hindlimbs after a walk, but he was still able to stand and walk; during the next day, dyspnea developed and the dog was immediately taken to the veterinarian. At arrival in the practice, he went into cardiac arrest, and although resuscitation was tried, he finally died. The owner reported that the dog was originally imported from Poland, and he confirmed that the dog had always been healthy and regularly vaccinated. As a result of the sudden and unexpected death of the subject, the owner requested a necropsy to identify the causes of death.

### 2.3. Histopathology

During necropsy, tissue samples for histological evaluation were fixed in 10% buffered formalin. Particular attention was paid to sampling the various muscles both affected by the pathology and those that were apparently normal. Diaphragm, esophagus, tongue, thigh (quadriceps femoral), and heart muscles were sampled. The quadriceps femoris muscle macroscopically looked normal. 

Tissue was fixed in 10% formalin for 24 h at room temperature, then it was embedded in paraffin and cut at a thickness of 4 microns. The sections were stained with hematoxylin and eosin (H&E) and Masson’s trichrome stain. 

### 2.4. Immunohistochemistry

Immunohistochemistry was performed using three antibodies against-dystrophin: C-terminus simultaneously specific for anti-human, -dog, and -mouse (corresponding to the 3′ end of the dystrophin gene); anti-human rod domain and anti-human N-terminus (corresponding to the 5′ end of the dystrophin gene). See specific technical data in Table S1 of supplementary material.

### 2.5. Transmission Electron Microscopy 

Samples were fixed in 1% OsO4 in cacodylate buffer, dehydrated in graded ethanol, and embedded in Araldite. Thin sections, stained with uranyl acetate and lead citrate, were examined using a Philips TEM CM100 Transmission Electron Microscope.

### 2.6. Whole-Genome Sequencing (WGS)

In order to investigate the underlying molecular basis, whole-genome sequencing (WGS), using genomic DNA isolated from the blood sample of the affected dog, was performed as described before [11]. Data corresponding to roughly 29× coverage of the genome were collected on an Illumina NovaSeq6000 instrument (2 × 150 bp). Read mapping, re-alignment, and variant calling were carried out as previously described [11] with respect to the CanFam3.1 genome reference assembly and the NCBI annotation release 105. The *DMD* gene representing the functional candidate was visually inspected for structural variants using Integrative Genomics Viewer [12]. WGS of the affected dog is available at the European Nucleotide Archive [13] sample accession SAMEA6249497. Numbering within the canine *DMD* gene as reported in the paper refers to the mRNA accession no. NM_001003343.1 and the protein accession no. NP_001003343.1.

## 3. Results

### 3.1. Necropsy Examination

**Figure 1:**
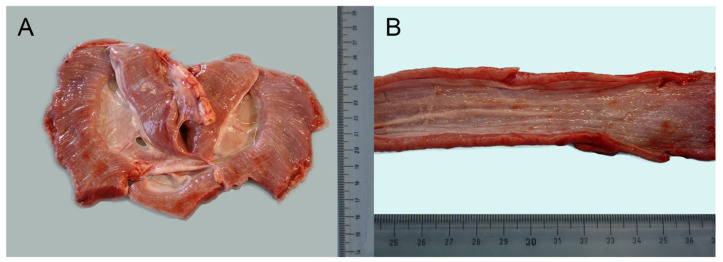
(**A**) Macroscopic image of diaphragm: it was severely thickened until 1 cm in section as well as the distal part of esophagus (4–5 mm thick) (**B**).

During necropsy, on external examination, the muscle conformation was normal without signs of muscle hypotrophy or hypertrophy and in a good state of nutrition. Interestingly, the diaphragm muscles were diffusely severely thickened, contracted with severe fibrosis, and the thickness of the muscle was about 1 cm (Figure 1A). In addition, the muscle of the base of the tongue and the distal part of esophagus were severely thickened (Figure 1B). The heart was increased in volume (cardiomegaly). As seen in the section, the right ventricular lumen was markedly dilated (dilated cardiomyopathy). The left and right atrioventricular valves were moderately swollen and edematous (valvular endocardiosis). Some sero-hemorrhagic fluid (around 10 mL) was found in the thoracic cavity with a severe acute pulmonary edema; the lungs showed multifocal petechiae and hemorrhages; whitish foam was found in the trachea and large bronchi. The sero-hemorrhagic fluid and the lung’s petechiae and hemorrhages could be due to resuscitation procedures.

### 3.2. Histopathology

**Figure 2:**
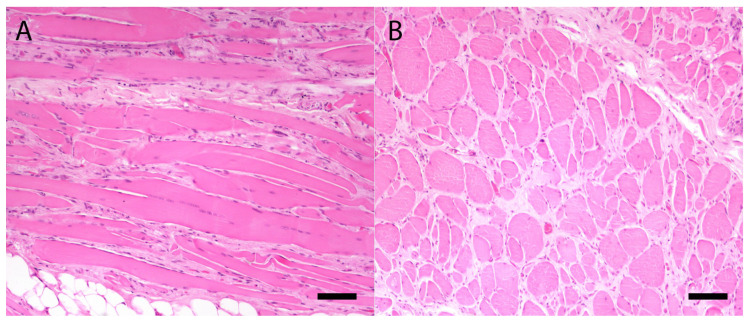
Longitudinal section of diaphragm stained with hematoxylin-eosin (H&E) (**A**), showing the rows of nuclei in the fibers (regeneration) and fiber splitting. (**B**). Cross-section of diaphragm stained with H&E showing marked atrophy in some muscle fibers and hypertrophy in others, and fiber splitting. A diffuse and mild fibrosis was also present. Bar = 100 micron.

All the muscles examined (diaphragm, esophagus, tongue, heart, thigh muscle) showed marked alterations. In the longitudinal sections, hematoxylin-eosin (H&E) rows of central nuclei in the fibers (regeneration) and fiber splitting (Figure 2A) were evident, and a moderate fibrosis was appreciated and confirmed with Masson’s trichrome stain. Cross-sections revealed marked variations in the fibers’ diameter with atrophy in some and hypertrophy in others (Figure 2B). Multifocal myofibers were fragmented and hypereosinophilic with a lack of a transversal band (coagulative necrosis) and internal nuclei and were multifocally infiltrated by lymphocytes and macrophages. There was also mild to moderate adipose tissue replacement, and fiber mineralization was rare. In the myocardium, there was slight fibrosis, whereas the lymphocytic and macrophage interstitial infiltrate was multifocal and moderate. A large focal area of coagulative necrosis was present.

### 3.3. Immunohistochemistry

**Figure 3:**
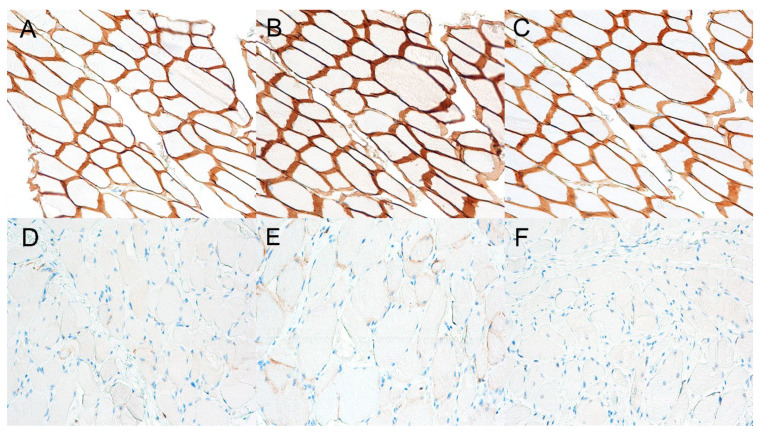
Control dog. Immunostains for the C-terminus (**A**), rod domain (**B**), and N-terminus (**C**) of the dystrophin in the diaphragm of a normal dog showing diffuse and strong sarcolemmal staining (positive control, transverse sections, objective 20×). In the affected dog, there was a total loss of positivity using both the anti-C-terminal (**D**) and N-terminal antibodies (**F**), and a very mild and multifocal signal with the anti-Rod antibody (**E**) (transverse sections, objective 20×).

All three antibodies tested for dystrophin gave a strong positivity and membrane positivity on the diaphragmatic muscle of a dog of the same breed used as a positive control (Figure 3A–C). On the contrary, all examined sections of the diaphragm, esophagus, tongue, and thigh muscle of the affected dog did not show anti-C-terminal (Figure 3D) and N-terminal (Figure 3F) antibody positivity, with the exception of some multifocal revertant fibers. There was weak and multifocal positivity to the anti-Rod antibody (Figure 3E).

### 3.4. Transmission Electron Microscopy

**Figure 4:**
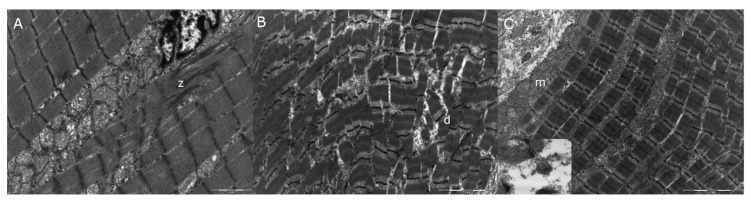
Electron microscopy of diaphragm muscle (**A**,**B**) and tongue (**C**): mild myopathic changes were seen as Z-line streaming (z), myofibrillar disarray (d), and mitochondrial hyperplasia (m): mitochondria seemed to be increased in size compared to the control (insert). 10,500×.

Transmission electron microscopy (TEM) analysis performed on the diaphragm and tongue muscle showed mild myopathic changes as Z-band streaming (Figure 4A) and myofibrillar disarray (Figure 4B) with mitochondria hyperplasia (Figure 4C).

### 3.5. Whole-Genome Sequencing 

**Figure 5:**
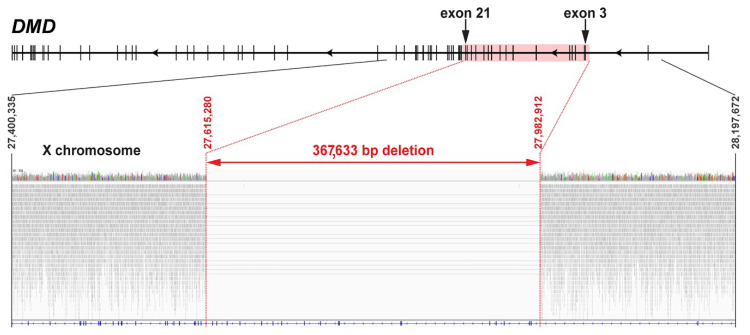
Large hemizygous partial deletion (CFAX g.[27,615,280_27,982,912del]) of the canine *DMD* gene in the Duchenne muscular dystrophy (DMD)-affected dog. The deletion spans exons 3–21 (highlighted by red box) and part of the flanking introns. The lower part shows an integrative genomics viewer screenshot of the region of interest from the whole-genome sequence data. Note the absence of mapped reads (shown in grey) within the region of 19 coding exons (red arrow). The light grey lines indicate read pairs, mapping across the breakpoints.

In the region of the *DMD* gene, a large hemizygous structural variant was detected in the genome of the affected dog (Figure 5). The variant encompasses a 367,633 bp region upstream of exon 3, up to part of intron 21. The deletion truncates the coding sequence of 19 of the 79 *DMD* exons annotated in the canine reference genome (exon 3 to exon 21). The formal variant designation is CFAX g.[27,615,280_27,982,912del]. No further single nucleotide variants affecting the coding regions of this gene were detected. Furthermore, the variant was absent from seven (five males, two females) publicly available WGS of Jack Russell Terriers as well as 576 dogs of 125 breeds and eight wolves [11].

## 4. Discussion

This report details a dystrophin-deficient 9-month-old Jack Russell Terrier with an abrupt onset of clinical signs that resulted in death the next day. The Polish owner reported that the affected dog had never shown any health problems before, and apart from the known Polish geographical origin, there was no pedigree information available. The juvenile age of death of this subject was in line with other cases of canine DMD reported previously, with a range from a few weeks to a few months of life [5,14,15]. The great majority of Golden Retriever Muscular Dystrophy (GRMD) dogs do not survive beyond age two [16]. Only some rare cases of similarly affected dogs of up to 5.6 years of age were reported before [17]. The clinical signs probably were not observed by the owner, but in any case, considering the conditions of the diaphragm, esophagus, tongue, and heart, we may suppose that it took place. It is obviously speculative, but we can assume the occurrence of heart failure, respiratory failure, and dysphagia.

It is reported that dystrophin deficiency results in progressive gross muscle atrophy in most breeds of dogs, but it causes a marked muscular hypertrophy in the mice, cats, and in dogs such as the Rat Terrier. Muscle fiber hypertrophy occurs, especially in early stages of the disorders, but extensive fiber necrosis in most cases leads to a general muscle atrophy. At this time, there is no explanation for this phenomenon, although it would appear that muscle hypertrophy is more apparent in animals of small stature [9].

Immunostaining in dystrophinopathies routinely involves the use of three antibodies targeting the different region of dystrophin, namely, the C- and N-terminus, as well as the central rod domain. In these cases, we tested all the three antibodies on formalin-fixed paraffin-embedded (FFPE) tissue of the case and one control dog. The results obtained in the affected dog were very similar to those observed in DMD-affected human patients [18], with total loss of positivity using both the anti-C-terminal and N-terminal antibodies, and a very mild and multifocal signal with the anti-Rod antibody. These results based on the use of these antibodies working on FFPE tissue are important in veterinary medicine, as it is not always possible to obtain fresh muscle biopsies and to then freeze them to send to suitable laboratories. Furthermore, the use of these antibodies allows the diagnosis of muscular dystrophy to be verified on the sample available to the pathologist. It is also possible to work retrospectively on archived samples. The key tests performed on the biopsy sample for muscular dystrophies were immunohistochemistry and immunoblotting for dystrophin, and therefore, the presented immunohistochemistry findings in the studied Jack Russell Terrier are highly suggestive of the presence of a Duchenne-type disease phenotype.

The first identified case of canine muscular dystrophy was in a Golden Retriever in 1958. Since then, various muscular dystrophy phenotypes have been reported in at least 15 other breeds [19]. Therefore, the obtained genetic results confirmed the previous clinical and pathological suspicion of a Duchenne-type muscular dystrophy in the affected Jack Russell Terrier. Different but similar gross deletions have been reported before in the Duchenne type of muscular dystrophy in dogs encompassing the entire *DMD* gene (OMIA 001081-9615) [2,15]; or, similarly to the findings in the herein presented dog, affecting a significant number of the coding exons, as in an affected Tibetan Terrier showing a deletion encompassing *DMD* exons 8 to 29 [20]. Other previously reported *DMD*-associated loss-of-function variants causing canine Duchenne-like muscular dystrophies include structural variants such as smaller sized deletions or gross insertions, as well as variants affecting single or smaller numbers of nucleotides such as splicing variants, inversions, or nonsense (stop-gain) variants (OMIA 001081-9615). 

## 5. Conclusions

The herein reported partial deletion of approximately a fifth of the canine *DMD* gene leads to a true null allele of the *DMD* gene, with the lack of protein expression being experimentally confirmed. The identified deletion variant might have occurred de novo during meiosis of the maternal germ cells and subsequently passed to the offspring as a consequence of low-level mosaicism in the mother, or it may have been transmitted as an X-linked recessive variant on the maternal path within the family of the affected dog. No evidence for further cases was obtained; we can, therefore, speculate that the mutation might also have occurred post-zygotically during early embryonic development of the affected dog explaining this single case. Taken together, this is the first report of a *DMD*-associated Duchenne-like muscular dystrophy in the Jack Russell Terrier breed. This study provides an example of a pathogenic disease-causing variant underlying a sporadic syndrome observed in dogs. Precision medicine using WGS has been proven to be suitable in recent years to help diagnose rare diseases in routine diagnoses in veterinary medicine: to both confirm genetic etiology and to obtain an insight into the molecular mechanisms involved. Nonetheless, the Golden Retriever muscular dystrophy continues to be the best-documented of the canine dystrophinopathies [21]. Thus far, there have been reports of 12 different canine breed-specific DMD-associated variants in the *DMD* gene (OMIA 001081-9615). The herein described phenotype of the affected Jack Russell terrier was most likely caused by the identified large genomic ~368kb deletion, spanning 19 coding exons of the *DMD* gene. This partial deletion truncates 18% of the coding sequence from exon 3 to 21 from the 79 exons annotated in the canine reference genome.

## Supplementary Materials

The following are available online at https://www.mdpi.com/2073-4425/11/10/1175/s1, Table S1. The table shows the technical specifications of the 3 antibodies used to highlight the various parts of dystrophin.
