# Supplementary material for: X-Linked Duchenne-Type Muscular Dystrophy in Jack Russell Terrier Associated with a Partial Deletion of the Canine DMD Gene
Source: Genes (Basel). 2020 Oct 8;11(10):1175. doi: 10.3390/genes11101175 (PMC7600251; doi:10.3390/genes11101175)
Supplement: Supplementary file 1 [file genes-11-01175-s001.pdf]

**Table S1.** The table shows the technical specifications of the 3 antibodies used to highlight the various parts of dystrophin.

| Specifity | Clone | Company | Dilution | Antigen recovery |
|---|---|---|---|---|
| Dystrophin C-terminus | polyclonal | abcam | 1:100 | Heat 52 min at 95° C, Tris EDTA pH 9 |
| Dystrophin Rod-domain | 13H6 | abcam | 1:30 | Heat 32 min at 95° C, Tris EDTA pH 9 |
| Dystrophin N-terminus | 34C5 | Novocastra | 1:20 | Heat 36 min at 95° C, Tris EDTA pH 9 |
